# Role of COX-2/mPGES-1/Prostaglandin E2 Cascade in Kidney Injury

**DOI:** 10.1155/2015/147894

**Published:** 2015-02-01

**Authors:** Zhanjun Jia, Yue Zhang, Guixia Ding, Kristina Marie Heiney, Songming Huang, Aihua Zhang

**Affiliations:** ^1^Nanjing Key Laboratory of Pediatrics, Nanjing Children's Hospital Affiliated to Nanjing Medical University, Nanjing 210008, China; ^2^Department of Nephrology, Nanjing Children's Hospital Affiliated to Nanjing Medical University, Nanjing 210008, China; ^3^Department of Internal Medicine, University of Utah, Salt Lake City, UT 84132, USA

## Abstract

COX-2/mPGES-1/PGE2 cascade plays critical roles in modulating many physiological and pathological actions in different organs. In the kidney, this cascade is of high importance in regulating fluid metabolism, blood pressure, and renal hemodynamics. Under some disease conditions, this cascade displays various actions in response to the different pathological insults. In the present review, the roles of this cascade in the pathogenesis of kidney injuries including diabetic and nondiabetic kidney diseases and acute kidney injuries were introduced and discussed. The new insights from this review not only increase the understanding of the pathological role of the COX-2/mPGES-1/PGE2 pathway in kidney injuries, but also shed new light on the innovation of the strategies for the treatment of kidney diseases.

## 1. Introduction

Kidney diseases including chronic kidney disease (CKD) and acute kidney injury (AKI) are rapidly increasing worldwide. Nowadays CKDs are affecting more than 10% of world population [1]. The incidence of AKI varies from 140 to 620 per million people based on different reports and is becoming a global concern [2, 3]. Recently, although the understanding on CKD and AKI was substantially updated with the extensive studies in the fields, the therapeutic outcomes of CKDs and AKI are still unsatisfactory. In recent decades, the ACEI/ARB-based therapy constantly dominates doctors' prescription for CKD treatment with no significant renovation. However, ACEI/ARB did not impede the progression of CKDs in a number of CKD patients. In AKI patients, the progression and application of intensive therapy did not dramatically change the therapeutic outcomes of this population. AKI patients in intensive care unit (ICU) still suffered very high in-hospital mortality rates by 40–60% and prolonged hospital stays [4]. This unacceptable situation chiefly resulted from the incomplete understanding of the pathogenesis of CKD and AKI, leading to the lack of effective therapeutic targets.

Among the contributors of kidney injury, inflammation is the established causative factor in both CKD and AKI [5–8]. As an important inflammatory mediator, PGE2 has drawn a lot of attention in the research of kidney disease [9–11]. The kidney is a region with abundant production of prostaglandins including PGE2, PGI2, PGD2, PGF2*α*, and TXA2 [12]. Among these prostaglandins, PGE2's effects on renal pathophysiology were best characterized [12, 13]. Under the physiological conditions, renal PGE2 importantly contributes to fluid metabolism and blood pressure regulation [9, 12]. Under the pathological states, PGE2 mostly mediates the renal injury induced by various insults [10, 11, 13–15]. In general, PGE2 functions through binding four G-protein-coupled receptors designated EP1, EP2, EP3, and EP4 [13, 16]. EP2 and EP4 act via stimulating cAMP signaling, whereas EP3 exerts activity by inhibiting cAMP production [13, 16]. EP1 is distinct from other EP receptors and functions via a calcium-dependent signaling pathway [13, 16]. In the kidney, EP4 and EP1 have been shown to play a detrimental role in mediating the diabetic and nondiabetic glomerular injuries [13, 15, 17, 18]. In contrast, a recent report demonstrated an antifibrotic effect of EP4 receptors in a unilateral ureteral obstruction (UUO) mouse model [19]. As for the roles of EP receptors in controlling the fluid metabolism in kidney, EP1 has an established role in exerting natriuresis and diuresis [12, 16, 20–22]. However, the renal effects of other EP receptors on fluid control are still elusive. In this review, we will fully introduce the advance of the COX-2/mPGES-1/PGE2 cascade in the pathogenesis of kidney injury. Particularly mPGES-1, the best characterized PGE2 synthase and most promising target of new anti-inflammatory drugs, will be emphasized.

## 2. PGE2 and PGE2 Synthases

PGE2 is generated through a COX/PGE2 synthases cascade [23–25]. As the upstream components of this cascade, COX-1 and COX-2 chiefly regulate the production of five major prostaglandins of PGE2, PGI2, PGD2, PGF2*α*, and TXA2 via adjusting the substrate (PGH2) supply to the individual prostaglandin synthetic enzymes [23, 26]. COX-1 is constitutively expressed with greater levels in platelets and gastric epithelial cells [27–29]. In platelets, COX-1-mediated TXA2 release not only plays a key role in platelets activation and aggregation, but also leads to vasoconstriction. In gastric epithelial cells, COX-1 is cytoprotective against injuries by promoting PGE2 production [30]. COX-2 is an inducible enzyme, particularly under inflammatory states [23, 30]. Selective COX-2 inhibitors have been widely used in clinic as fever relievers and pain killers. In the kidney, COX-2 has a relatively high expression and plays important roles in regulating renin release [24, 25, 31–33], water/salt metabolism [24, 25, 31, 33, 34], and kidney development [24, 35, 36]. More importantly, growing evidence demonstrated a detrimental role of COX-2 in mediating kidney injuries [10, 14, 37, 38].

Currently, three PGE2 synthases had been cloned including microsomal prostaglandin E synthase-1 (mPGES-1), mPGES-2, and cytosolic PGES (cPGES). Among the three PGESs, mPGES-1 has been identified to have the best PGE2 synthetic property in both in vitro and in vivo conditions [39–41]. In general, the baseline mPGES-1 expression is relatively low in various cell types and organs [42] but is highly inducible in response to physiological or pathological stimuli [42, 43]. mPGES-2 and cPGES are constitutively expressed and had been thought to be responsible for the baseline PGE2 production [43, 44]. However, both mPGES-2 and cPGES did not display the convincing capability of PGE2 production in animals with genetic disruption [43, 45–47] in spite of several in vitro reports showing their activities in generating PGE2 [48–50]. Mice lacking cPGES are perinatally lethal and the reduction of lung PGE2 content in cPGCS-null embryos is accompanied with a global decrease of prostanoids (PGE2, TXB2, and PGI2) and prostanoid-generating enzymes (COX-1, COX-2, and cPLA2), suggesting that such changes of PGE2 and other prostanoids might be secondary to a developmental defect [45, 46]. Recently, a number of evidences from our and other groups convincingly demonstrated that mPGES-1 is a key enzyme in controlling both baseline and induced PGE2 levels [10, 11, 14, 39, 41]. Moreover, mPGES-1-derived PGE2 has been shown to be very important in modulating a series of physiological and pathological activities in animals. For example, we found that mPGES-1-derived PGE2 is critical in regulating renal handling of water and salt in response to water/salt overload or water depletion [39, 51–55]. In addition, mPGES-1-derived PGE2 also exhibited antihypertensive effects possibly via countering RAS system in kidney and vasculatures [41, 56, 57]. More importantly, mPGES-1 also contributes to chronic and acute kidney injuries [10, 11], suggesting a potential target in treating kidney diseases in patients.

## 3. mPGES-1 and CKD (Diabetic and Nondiabetic CKD)

### 3.1. mPGES-1 in Diabetic Kidney Injury

Urinary PGE2 production is elevated in diabetes mellitus [14] and mediates diabetes-associated glomerular disease as evidenced by the attenuation of glomerular damage following the antagonism of PGE2 receptors [15, 18]. mPGES-1, as the best-characterized PGE2 synthase, is supposed to be the enzymatic source of renal PGE2 generation and could be involved in the occurrence and development of diabetic kidney injury. To verify this hypothesis, we treated mPGES-1 WT and KO mice with STZ to induce a type-1 diabetic model and evaluated the renal PGE2 production and glomerular injury [14]. In this experimental setting, mPGES-1 deletion did not influence the blood glucose level in diabetic mice in comparison to the WT controls, indicating a paralleled onset of diabetes and similar pancreatic *β*-cell damage. This finding agrees with the report showing that mPGES-1 has no effect on islet in response to IL-1*β* treatment [58]. Unexpectedly, neither renal PGE2 production nor glomerular injury was affected by mPGES-1 deletion following 6-week diabetes induced by STZ [14]. In addition, mPGES-2, cPGES, and 15-PGDH in the kidney were also unaltered by diabetes, suggesting that an unknown PGE2 synthetic enzyme might be attributable to the PGE2 production and the pathological activity in this process [14]. Moreover, these results also suggested that hyperglycemia is not an activator of the mPGES-1/PGE2 cascade in diabetic kidney. Surprisingly, in obese db/db mice with type-2 diabetes, mPGES-1 was significantly induced in glomeruli [59]. Suppression of mPGES-1 by a PPAR*γ* agonist rosiglitazone significantly blunted urinary PGE2 output [59]. This discrepancy in mPGES-1 regulation between two models could be due to the diverse pathogenic mechanisms. For example, the absence of the leptin receptor itself, obesity, more severe disorders of lipid metabolism, and hypertension in db/db mice all could be attributable to the distinct regulation of mPGES-1. Overall, the preceding evidence does not demonstrate any specific PGE2 synthase in mediating renal PGE2 production in mice with type-1 diabetes. More evidence in human subjects and other animal species is definitely needed to further clarify the renal role of mPGES-1 and other PGE2 synthases in type-1 diabetic condition. Additionally, whether mPGE-1 plays a role in kidney injury of type-2 diabetes also requires further studies.

### 3.2. mPGES-1 in Nondiabetic CKD

CKD progresses into the end stage renal disease in a number of patients. To prevent or retard the progression of CKD via an effective therapy is of critical importance in clinic. However, the clinical evidence from ACEI/ARB-based first-line treatment indicated that such an intervention merely slows but cannot stop the CKD progression. Therefore, to innovate and strengthen the strategies of CKD therapy is an urgent task for both clinicians and researchers.

PGE2 is a known mediator of inflammation contributing to the pathogenesis of many diseases including kidney injury [13, 24, 60–63]. As the best-characterized specific PGE2 synthase, mPGES-1 has drawn a lot of attention in the kidney disease field and was investigated in a 5/6 nephrectomy mouse model by our group [11]. Following four weeks of 5/6 nephrectomy, the mPGES-1 WT mice developed significant renal failure as evidenced by the increased blood urea nitrogen and creatinine, robustly reduced creatinine clearance, disorders of calcium phosphorus metabolism, and anemia. In line with the loss of renal function, the WT mice also displayed proteinuria, glomerular morphological lesions, and the substantial loss of podocytes. Interestingly, all these abnormalities were strikingly improved in the mice with mPGES-1 invalidation except for the anemia with an aggravation in KO mice. The aggravated anemia was evidenced by greater reduction of Hct and the markedly increased spleen weight. Such a phenomenon of deteriorated anemia is possibly due to the impaired compensation of EPO production in the kidney [11]. Several studies indicated the stimulatory role of PGE2 in regulation of EPO production [64–67]. In mPGES-1 KO mice, the kidney PGE2 induction was remarkably blunted following 5/6 nephrectomy, which could account for the impaired EPO generation. However, this result cannot rule out the possible involvement of bone marrow in mediating the formation of erythroid colonies. In future studies, evaluating the effect of mPGES-1-derived PGE2 on erythroid colony formation is required to answer this concern.

Inflammation has a known role in mediating the development and progression of CKDs [6]. As expected, the inflammatory response was significantly attenuated in mPGES-1 KO mice with 5/6 nephrectomy, which is accompanied with the improvement of kidney morphology and function, suggesting that mPGES-1-mediated inflammation could be involved in the progression of CKDs. These findings were also supported by other reports demonstrating that inhibition of prostaglandins contributes to the protection of renal function in a renal ablation model [68, 69].

Renal concentrating abnormality is another known clinical complication in CKD patients with uncertain mechanisms. In mPGES-1 WT mice, increased urine volume and reduced urine osmolality were observed after kidney mass reduction. With mPGES-1 invalidation, such abnormalities were significantly improved in parallel with the induction of NKCC2 and AQP3 [11]. This agrees with the previous report showing that COX-2 inhibition potentiated renal concentrating capability in rats with renal mass ablation [69]. These findings highly suggested that mPGES-1-derived PGE2 mediates urine concentrating defect in CKDs at least to some extent.

Emerging evidence from mPGES-1 KO mice revealed a new contributor of mPGES-1 in modulating the renal function in nondiabetic CKDs. Moreover, the findings also provided the therapeutic potential of mPGES-1 inhibitors for the treatment of CKD. However, the side effect of anemia in patients with mPGES-1 inhibition could be a serious concern that needs to be dealt with. A combination of EPO with mPGES-1 inhibitors could be a suitable way in overcoming such an adverse effect.

## 4. mPGES-1 and AKI

AKI is a severe clinical complication with high mortality and morbidity [2]. Intervention of AKI is still a big challenge due to the complexity and poor understanding of pathogenesis of this disease [3]. AKI can result from direct or indirect insults acting on kidneys including all forms of shock, severe heart and liver diseases, ureteral obstruction, nephrotoxic drugs and toxins, and so on [2, 3]. As an established inflammatory mediator, PGE2 presumably plays a role in the development and progression of AKI. To test the possible role of PGE2 in AKI, mPGES-1, a specific PGE2 synthetic enzyme, was genetically disrupted in mouse, and several common insults including nephrotoxic drug cisplatin, LPS, and ischemia/reperfusion were applied to those mice [10]. In the cisplatin experimental setting, the mPGES-1/PGE2 cascade was strikingly stimulated in the renal tubular cells and deletion of mPGES-1 in mice resulted in a remarkable resistance to cisplatin-induced kidney injury possibly via the inhibition of the inflammatory response and oxidative stress [10]. However, mPGES-1 invalidation in mice did not protect renal function in the experimental settings of LPS and renal ischemia/reperfusion [10]. This discrepancy could be due to the difference of pathogenic mechanisms among those disease models. Indeed, we found a significant downregulation of mPGES-1 mRNA expression in the kidneys subjected to the ischemia/reperfusion [10] contrasting to a similar systemic inflammatory response in both genotypes following LPS administration, which suggested that mPGES-1 is not critical in mediating the inflammation in renal ischemia/reperfusion in this mouse strain. In consideration of the importance of the mPGES-1/PGE2 cascade in cancer development and progression [70, 71], mPGES-1 inhibition may play dual roles in the protection of kidney function and suppression of cancers in patients with cisplatin therapy.

## 5. COX-2 and CKD

COX-1 and COX-2 are upstream enzymes of the mPGES-1/PGE2 cascade via providing the substrate of PGH2 for PGE2 synthases (mPGES-1, mPGES-2, and cPGES) [9, 23]. COX-1 is constitutively and abundantly expressed [9, 23, 34]. In contrast, COX-2 serves as an inducible enzyme mediating the induction of various prostaglandins under the physiological and pathological conditions [9, 23]. In the kidneys, COX-2 is highly inducible in podocytes, mesangial cells, renal tubular epithelial cells, and interstitial cells [9]. Here we mainly introduce and discuss the role of COX-2 in CKDs.

### 5.1. COX-2 in Diabetic CKD

In diabetic condition, urinary PGE2 is elevated in parallel with the activation of COX-2 [14]. Convincing evidence from podocyte-specific COX-2 transgenic mice demonstrated a detrimental role of COX-2 in mediating diabetic glomerular injury possibly via modulating the prorenin receptor expression and inflammatory response in STZ-induced type-1 diabetes [72, 73]. More interestingly, Mohamed et al. reported that COX-2 induction in proximal tubules may contribute to the PGE2 production and diabetic proteinuria in a STZ mouse model [74]. Such an effect of tubule-originated PGE2 through COX-2 activation on inducing glomerular injury could be a paracrine action of PGE2. However, more evidence from animals with proximal tubule-specific COX-2 invalidation is needed to further validate such a notion. In the type-2 diabetic model including Zucker rats and db/db mice, kidney COX-2 expression was strikingly upregulated [59, 75]. Refecoxib, a specific COX-2 inhibitor, almost normalized the glomerulosclerosis and proteinuria in obese Zucker rats without affecting hyperglycemia [76], suggesting a pathogenic role of COX-2 in the kidney injury resulted from type-2 diabetes. All these findings highly suggested a therapeutic target of COX-2 in treating diabetic kidney disease. However, the known adverse effects of COX-2 inhibitors in the cardiovascular system, gastrointestinal tract, and kidney greatly limited its long term use in clinic. Considering the acceptable safety of long term use of low dose COX inhibitors in clinic, it is worthwhile to test the efficacy of low dose COX inhibitors for the treatment of diabetic kidney diseases via combination with RAS blockers and/or other therapies.

### 5.2. COX-2 in Nondiabetic CKDs

A number of studies performed in CKD models demonstrated the beneficial effect of COX-2 inhibitors in retarding the progression of kidney injury [37, 77–79]. In kidney ablation animal models, COX-2 inhibitors significantly attenuated proteinuria and structural damage and preserved better renal function [77]. Emerging evidence also indicated a comparable efficacy of COX-2 inhibition on kidney protection as ACEI treatment, independently of blood pressure control [80]. Combination of ACEI and a COX-2 inhibitor exhibits additive effect in ameliorating the proteinuria in SHR rats with hypertensive kidney injury [78] and glomerular sclerosis in rats with renal mass reduction [77]. In podocytes, COX-2 is detrimental in mediating the podocyte injury and subsequent glomerular damage and renal dysfunction [73, 81]. The authors also demonstrated that deletion of thromboxane receptor but not PGE2 receptor EP4 led to a striking attenuation of albuminuria and podocyte foot process effacement in a chronic kidney disease model induced by Adriamycin [73]. For the activation of COX-2 in those pathological processes, proinflammatory cytokines (e.g., interleukin-1 and TNF-*α*) and reactive oxygen species (ROS) have been reported to play critical roles [82]. Generally, COX-2 and other inflammatory signaling pathways form a positive feedback loop to promote the development and progression of disease status [10, 82]. In addition, activation of COX-2 also positively enhances the activity of COX-2 itself via its end metabolites like prostaglandins [83]. Ultimately, a collection of solid evidence from animal studies strongly supports a pathogenic role of COX-2 in many forms of CKDs. Targeting COX-2 could be a promising strategy in treating this kind of disease. However, the nonselective antagonism of COX-2 inhibitors on its downstream metabolites resulted in some severe side effects in the clinic, which greatly limited its recruitment in the therapeutic strategies of CKDs.

## 6. COX-2 and AKI

In the mammalian kidneys, the major nephron sites expressing COX-2 include cortical thick ascending limb, macula densa, medullary interstitial cells, and the endothelium of arteries and veins and glomerular podocytes. The toxicity of COX inhibitors on kidney was first identified in the 1950s. Although the occurrence of COX inhibitors-associated nephrotoxicity is low (1–5%), it is still common owing to the widespread use of this kind of drug. Most cases with COX inhibitor-associated acute kidney injury were found to have additional risk factors of AKI including CKDs, congestive heart failure (CHF), hypertension, chronic liver disease, plasma volume depletion, and use of loop diuretics [4, 84–90]. Due to the known role of COX-2 and its end metabolites in regulating renal physiology, we can reasonably conceive that patients with AKI risk factors may need active COX-2 signaling in setting up a new balance of renal function in fluid excretion, intrarenal hemodynamics, et al. Once this balance was disrupted by inhibiting COX-2 signaling, the AKI risk factor(s) may get the upper hand in the abnormal kidneys and promote AKI occurrence. Therefore, avoiding application of COX-2 inhibitors in patients with AKI risk factors is of vital importance in lowering the incidence of COX inhibitor-related AKI.

Although the use of COX-2 inhibitors is associated with AKI incidence under some conditions, COX-2 inhibition also displayed beneficial roles in some forms of AKI. For example, cisplatin-induced AKI was attenuated by selective COX-2 inhibitors [10]. In this particular disease model, COX-2-associated inflammation may serve as the key factor in mediating the kidney injury. However, in the ischemia/reperfusion (I/R) kidney injury, COX-2/prostaglandins may play an important role in counteracting the reduction of glomerular filtration rate to maintain a better renal function. For example, some reports indicated that suppression of COX-2 activity in I/R kidneys results in a deterioration of renal injury [91, 92]. However, Feitoza et al. reported that indomethacin, a nonselective COX inhibitor, protected against I/R-induced kidney injury in rats [93]. Ranganathan et al. also demonstrated that COX-2-derived PGE2 promotes I/R-induced kidney injury via EP4 receptor [38]. The discrepancy derived from different research groups is possibly due to the inconsistency of the experimental approaches such as different time duration of ischemia, difference of animal strain or species, and selection of COX inhibitors with COX-independent properties. Due to the complexity and diversity of the pathogenic mechanisms in AKI, the outcome of COX-2 interruption may be variable among different types of AKI. Overall, the effects of COX-2 inhibitors on kidneys might depend on the pathogenic mechanisms of kidney diseases or the difference of pathological insults.

## 7. Perspectives

The important roles of COX-2/mPGES-1/PGE2 signaling in controlling the renal physiology and pathology have been demonstrated. The potential of clinical application of COX-2 inhibitors was limited by their adverse effects seen in the cardiovascular system, GI system, and kidneys. The most plausible reason for the adverse effects from COX-2 inhibitors is because of its nonselective activity on inhibiting the downstream metabolites, particularly the protective prostaglandins (e.g., PGI2). Therefore, selective inhibition of the guilty prostanoid by inactivation of the specific downstream enzyme of the COX-2 signaling pathway is a novel and promising strategy in treating the kidney diseases including CKDs and AKIs. By now, a number of mPGES-1 inhibitors have been generated and some of them are currently in the clinical trials. We have more confidence in the safety and efficacy of this kind of new anti-inflammatory drugs for the treatment of kidney diseases, as well as the diseases of other organs, by specifically targeting mPGES-1.
